# Dataset on the Acceptance of e-learning System among Universities Students' under the COVID-19 Pandemic Conditions

**DOI:** 10.1016/j.dib.2020.106176

**Published:** 2020-08-18

**Authors:** Manaf Al-Okaily, Hamza Alqudah, Ali Matar, Abdalwali Lutfi, Abdallah Taamneh

**Affiliations:** a School of Business, Jadara University, 733, Irbid, Jordan.; b College of Business Administration, King Faisal University, 81932, Al-Alahsa, Saudi Arabia.; c College of Business Administration, Ajloun National University, 26810, Ajloun, Jordan.

**Keywords:** Distance learning, e-learning system, COVID-19, Corona virus, Acceptance, Jordan universities, TAM model

## Abstract

The COVID-19 pandemic has produced an unprecedented change in the educational system worldwide. Besides the economic and social impacts, there is a dilemma of accepting the new educational system "e-learning" by students within educational institutions. In particular, universities students have to handle several kinds of environmental, electronic and mental struggles due to COVID-19. To catch the current circumstances of more than two hundred thousand Jordanian university student during COVID-19. The students have been randomly selected to respond on an online survey using universities' portals and websites between March and April 2020. At the end of the data gathering process, we have received 587 records. The dataset includes 1) Demographics of students; 2) students’ perspectives concerning the factors influencing their intention to use e-learning system within the Jordanian universities context. Data were analyzed using Partial Least Squares - Structural Equation Modelling (PLS-SEM). Next, the result has confirmed the positive of direct effect variables (subjective norm, perceived ease of use, and perceived usefulness) on the students’ intention to use e-learning system. Next, the result has also confirmed the mediating effect of perceived usefulness and perceived ease of use between subjective norm and the behavioral intention to use the e-learning system with partially supported.

**Specifications Table**

**Table:** 

**Subject**	Education, Education Management
**Specific subject area**	The Jordanian universities students’ intention to use e-learning system during the COVID-19 pandemic, subjective norm (SN), perceived ease of use (PEU), and perceived usefulness (PU), technology acceptance model (TAM).
**Type of data**	The raw data is available in Excel Workbook format. The analysed data in this article are provided in tables and figures.
**How data were acquired**	Online Survey
**Data format**	Raw
**Parameters for data collection**	The target population of this work was Jordanian universities students who are affected by COVID-19 pandemic. In light of the universities closure, all Jordanian universities have converted to the e-learning system. as a result, more than 200 thousand students from various universities were required to handle with a new educational system unprecedentedly.
**Description of data collection**	We colleted data using an online survey through universities' portals and websites between March and April 2020. The participants of this dataset were the Jordanian universities students. In this regard, the questionnaire is provided as a supplementary file
**Data source location**	Jordan
**Data accessibility**	Repository name: Mendeley Data Data identification number: 1 Direct URL to data: https://bit.ly/3i8KkI5.

**Value of the Data**

• The dataset is valuable because it can be utilized as a reference for understanding the effect of factors, namely: subjective norms, perceived ease of use and perceived usefulness on the student's intention to accept the e-learning system.
• This data presents the natural flow to measure students’ intention to acceptance/use the e-learning system during COVID-19 pandemic, which can be replicated in other countries.
• This data can help to understand the factors that affect the e-learning system acceptance through integrating subjective norms with the extended Technology Acceptance Model (TAM). Besides, using both variables: perceived ease of use and perceived usefulness as mediation between subjective norms and the e-learning system acceptance.
• Finally, the data is useful for all parties involved, especially for universities management, ministry of higher education, decision-makers in a country, researchers and practitioners in the e-learning system.

## 1 Data description

The data presented in this paper is focused on the students in the Jordanian universities who using the e-learning system. The research was conducted according to and complies with all regulations established in the ethical guidelines by the Jordanian Ministry of Higher Education and Scientific Research. The data file spreadsheet accompanying this article consists of 587 rows and 24 columns of dataset. Every row represents an individual's response to a survey. A five-point range scale was applied to allow the respondents to indicate how much they disagree or agree with a certain statement, so a numerical value in the dataset file means the respondent level of agreement, with 1 being "strongly disagree" and 5 being "strongly agree". Demographic dataset regarding Jordanian universities students’ profile indicated that 394 were male and 193 were female. Regarding the age 148 (25%) of the respondents were between 18 and 20 years old, 311 (53%) of the respondents were between 21 and 23 years old, and 128 (22%) of the respondents were more than 23 years. The majority of respondents were bachelor students 572 (97%) followed by master degree 15 (3%). Of the 587 students that participated in the study, 111 are from the Faculty of Business, 172 from the Faculty of Languages and Arts, 39 from the Faculty of Engineering, 116 from the Faculty of Pharmacy, 125 from the Faculty of Educational Sciences, and 24 from the Faculty of Law. Further, each variable's items in the questionnaire was given a label, as shown in Table 1.

**Table 1:** Iteme Loading (IL), Composite Reliability (CR), and Average Variance Extracted (AVE).

Construct Name		Items Name	IL	CR	AVE
Behavioral Intention (BI)		BI1	0.924		0.864
		BI2	0.932		0.87
		BI3	0.93		
		BI4	0.946		
Perceived Ease of Use (PEU)		PEU1	0.893	0.932	0.774
		PEU2	0.924		
		PEU3	0.883		
		PEU4	0.818		
Perceived Usefulness (PU)		PU1	0.888	0.929	0.768
		PU2	0.918		
		PU3	0.785		
		PU4	0.907		
**Construct Name**		**IN**	**IL**	**CR**	**AVE**
**Second-Order**	**First-Order**				
Subjective Norms (SN)	Social Influence (SI)	SI1	0.843	0.883	0.653
		SI2	0.828		
		SI3	0.807		
		SI4	0.752		
	Peer Influence (PI)	PI1	0.907	0.847	0.818
		PI2	0.901		
		PI3	0.923		
		PI4	0.888		

Wherein, the majority of respondents were undergraduate students (572). Of the 587 students that participated in the study, 172 from the Faculty of Languages and Arts, 116 from the Faculty of Pharmacy, 111 are from the Faculty of Business, 77 from Faculty of Science and Information Technology, 48 from Faculty of Educational Sciences, 39 from the Faculty of Engineering, and 24 from the Faculty of Law.

Table 1. shows the test of measurement model (inner model) including composite reliability, indicators reliability, average variance extracted. In regard to the composite reliability, the criterion of composite reliability was assessed to verify the internal consistency reliability. The values showed the constructs scores are at acceptable level of reliability [[2], [3]]. Hence, internal consistency was confirmed. For the factors' loading, all items were higher than 0.70. Besides, the validity of the instrument was proved by calculating the average variance extracted [1]. Wherein, the results of the average variance extracted (convergent validity) are at acceptable level, which all variables have average variance extracted value larger than 0.50 (see Table 1).

In addition, as shown in Table 2 the test of discriminant validity was also a part of measurement model (inner model) assessments. The discriminant validity was conducted to evaluate the range to which a provided study latent variable is distinct from others. Hence, when the average variance extracted of an individual latent construct is higher than the multiple squared correlations of that construct with other constructs, the discriminant validity will be at an acceptable level [4]. Thus, the results revealed that all studied variables had good discriminant validity values (see Table 2).

**Table 2:** Fornell–Larcker criteria (The AVE square root).

	BI	PI	PEU	PU	SI
Behavioral Intention	**0.933**				
Peer Influence	0.641	**0.905**			
Perceived Ease of Use	0.774	0.636	**0.88**		
Perceived Usefulness	0.812	0.685	0.837	**0.876**	
Social Influence	0.667	0.764	0.648	0.693	**0.808**

**Note:** The square root of the AVE values shown in bold represent.

## 2 Experimental design, materials and methods

Data were gathered using online survey through Jordanian universities students’ portals and websites (between March and April 2020). The students were asked to fill in the online questionnaire through the provided link. From those students, there were 587 responses. The questionnaire and the answers to the questions are provided as a supplementary file. The data were analysed using statistical test including PLS-SEM approach. We used Smart PLS 3.0 software [1]

The study's model was contained two level of constructs (upper and lower), thus to conduct the measurement model assessment, each of composite reliability, indicators reliability, average variance extracted, and discriminate validity were tested. In regard to the composite reliability, the criterion of composite reliability was assessed to verify the internal consistency reliability. The values in Table 1 showed the constructs scores exceed the acceptable level of reliability 0.7 [[2], [3]] (see Table 1). Hence, internal consistency was confirmed. Besides, all factors' loading is higher than 0.70. The validity of the instrument was proved by calculating the average variance extracted [1]. Wherein, the average variance extracted is the indicator used for measuring convergent validity, by measuring the variance value of that the items share with their respective variable [1]. The results of the average variance extracted (convergent validity) are also presented in Table 1, which all variables have average variance extracted value larger than 0.50.

Finally, the discriminant validity test as shown in Table 2 was also conducted to evaluate the range to which a provided study latent variable is distinct from others. Whereby, when the average variance extracted of an individual latent construct is higher than the multiple squared correlations of that construct with other constructs, the discriminant validity will be at an acceptable level [4]. As illustrated in Table 2, all studied variables had good discriminant validity values.

## Ethics statement

This work neither involves chemicals, procedures or equipment that have any unusual hazards inherent in their use nor involves the use of animal or human subjects.

## Declaration of Competing Interest

The authors declare that they have no known competing financial interests or personal relationships which have, or could be perceived to have, influenced the work reported in this article.
